# U-shaped association between serum calcium and in-hospital mortality in diabetes patients with congestive heart failure: a cohort study

**DOI:** 10.1038/s41598-024-63603-w

**Published:** 2024-06-11

**Authors:** Kai Zhang, Tianqi Zhang, Qian Yu Lv, Yu Han, Tianyi Cai, Fang ming Gu, Zhao xuan Gu, Jia Yu Zhao, Jia Ying liang, Min Gao, Ya Fang Gao, Rui Hu, Dan Cui, Bo Li, Kexiang Liu

**Affiliations:** 1 Cardiovascular Surgery Department of Jilin University Second Hospital, No. 218, Ziqiang Street, Changchun, Jilin Province China; 2 Department of Ophthalmology, First Hospital of Jilin University, Changchun, China; 3 Department of Cancer Center, The First Hospital of Jilin University, Changchun, China; 4 Department of Ophthalmology, Second Hospital of Jilin University, Changchun, China

**Keywords:** Blood calcium, In-hospital mortality, Diabetes, Association, Cubic spline function model, Subgroup analysis, Diseases, Endocrinology, Medical research, Risk factors

## Abstract

Previous studies have reported that the significant association between serum calcium and mortality substantially in patients, especially among those with intensive care unit (ICU). And In diabetes mellitus, congestive heart failure (CHF) is a significant comorbidity. We aim to evaluate the association between serum calcium levels and in-hospital mortality among patients with diabetes and congestive heart failure. The participants in this study were extracted from the Medical Information Mart for Intensive Care IV (MIMIC-IV) database. To scrutinize potential associations between serum calcium levels and in-hospital mortality, a comprehensive analysis encompassing multivariate logistic regression, cubic spline function model, threshold effect analysis, and subgroup analysis was performed. This retrospective cohort study encompassed 7063 patients, among whom the in-hospital mortality stood at 12.2%. In the multivariate logistic regression, adjusted odds ratios (ORs) were contrasted with the reference category Q6 (8.8–9.1 mg/dL) for serum calcium levels and in-hospital mortality. The adjusted ORs for Q1 (≤ 7.7 mg/dL), Q2 (7.7–8 mg/dL), and Q7 (≥ 9.1 mg/dL) were 1.69 (95% CI 1.17–2.44, p = 0.005), 1.62 (95% CI 1.11–2.36, p = 0.013), and 1.57 (95% CI 1.1–2.24, p = 0.012) respectively. The dose–response analysis uncovered a U-shaped relationship between serum calcium levels and in-hospital mortality in diabetic patients with heart failure. Subgroup analyses confirmed result stability notwithstanding the influence of diverse factors. Our investigation revealed a U-shaped correlation between serum calcium levels and in-hospital mortality in diabetes patients with congestive heart failure, pinpointing a significant inflection point at 9.05 mg/dL.

## Introduction

Diabetes mellitus (DM) encompasses a diverse spectrum of conditions characterized by hyperglycemia, categorized as type 1, type 2, “other specified types,” and gestational diabetes mellitus^1^. The prevalence of DM has surged markedly in recent decades, evolving into a significant global health challenge^2^. According to the International Diabetes Federation (IDF) Atlas 10th edition, the projection indicates that the diabetic population will escalate to 643 million by 2030 and 783 million by 2045^3,4^. Congestive heart failure (CHF), a predominant and severe cardiovascular consequence of DM, has garnered substantial global attention^4^. Around 40% of CHF patients are afflicted with DM^5^, which also entails a two- to five-fold elevation in heart failure risk compared to normoglycemic individuals^6^. Previous inquiries underscore the unfavorable clinical prognosis for diabetic patients with heart failure, exhibiting higher mortality rates than their normoglycemic counterparts^7,8^. The advent of heart failure in diabetic individuals precipitates a tenfold surge in mortality rates, resulting in a meager five-year survival rate of 12.5%^9^. Consequently, an urgent imperative arises to concurrently address DM and CHF. In recent years, the relationship between blood electrolyte disorder and the progress of diabetes and heart failure has gradually aroused the interest of scholars, especially blood calcium^10,11^.

Calcium is crucial for many physiological functions, including muscle contraction, secretion of neurotransmitters and hormones, blood coagulation, and mineralization of bone^12^. Approximately 50% of serum calcium is in the ionized form, while 40% in the bound form mainly to albumin, and 10% is bound to anions^13^. Serum total calcium is the total sum of three forms and is least affected by physiological changes or varieties in measurement. Therefore, total serum calcium is routinely used in clinical practice to represent calcium status in the human body^14^. An epidemiological study across multiple centers in the United States has indicated that calcium concentrations ≥ 2.38 mmol/l (9.5 mg/dl) correlate with heightened diabetes risk^15^. Substantial evidence suggests that elevated serum calcium levels contribute to heightened all-cause mortality among individuals with diabetes, primarily due to augmented risk of coronary artery calcification^16,17^. Elevated serum calcium levels are linked to increased vulnerability to heart failure with preserved ejection fraction in type 2 diabetes (T2D) patients^18^. Investigations by Jensen et al. have highlighted that both excessively high and low serum calcium levels in CHF patients are associated with elevated mortality rates^19^. Prior research underscores the detrimental impact of elevated blood calcium levels on diabetes management and associates extreme serum calcium levels with unfavorable heart failure outcomes. Significantly, heart failure and diabetes mellitus often co-occur, yet the simultaneous exploration of serum calcium levels in both conditions remains limited^5^.

To our knowledge, we are the first to evaluate such an association between serum calcium levels and patients with combined diabetes mellitus and heart failure, utilizing the first large-sample patient data from the U.S. context.

## Methods

### Data sources and study population

We conducted a retrospective cohort analysis utilizing the Medical Information Mart for Intensive Care IV (MIMIC-IV) database, a comprehensive compilation of contemporary electronic health records covering a decade of hospital admissions (2008–2019)^20^. MIMIC-IV, an updated iteration of MIMIC-III, contains extensive information encompassing patients’ demographics, laboratory results, medications, vital signs, surgical interventions, disease diagnoses, medication protocols, and post-discharge survival outcomes^21^. This dataset notably furnishes precise insights into mortality events, including in-hospital deaths and deaths within 90 days post-discharge^22^. Leveraging the MIMIC-IV database in-depth can offer valuable evidence for clinical decision-making and prognostic assessments. Research clearance for database access was granted by the institutional review bodies at the Massachusetts Institute of Technology and Beth Israel Deaconess Medical Center. Given the absence of clinical impact and the anonymization of protected health information, the necessity for consent was waived^23^. The responsible author (ID: 11639604) met the prerequisites for database access and assumed data extraction responsibilities.

**Figure 1:**
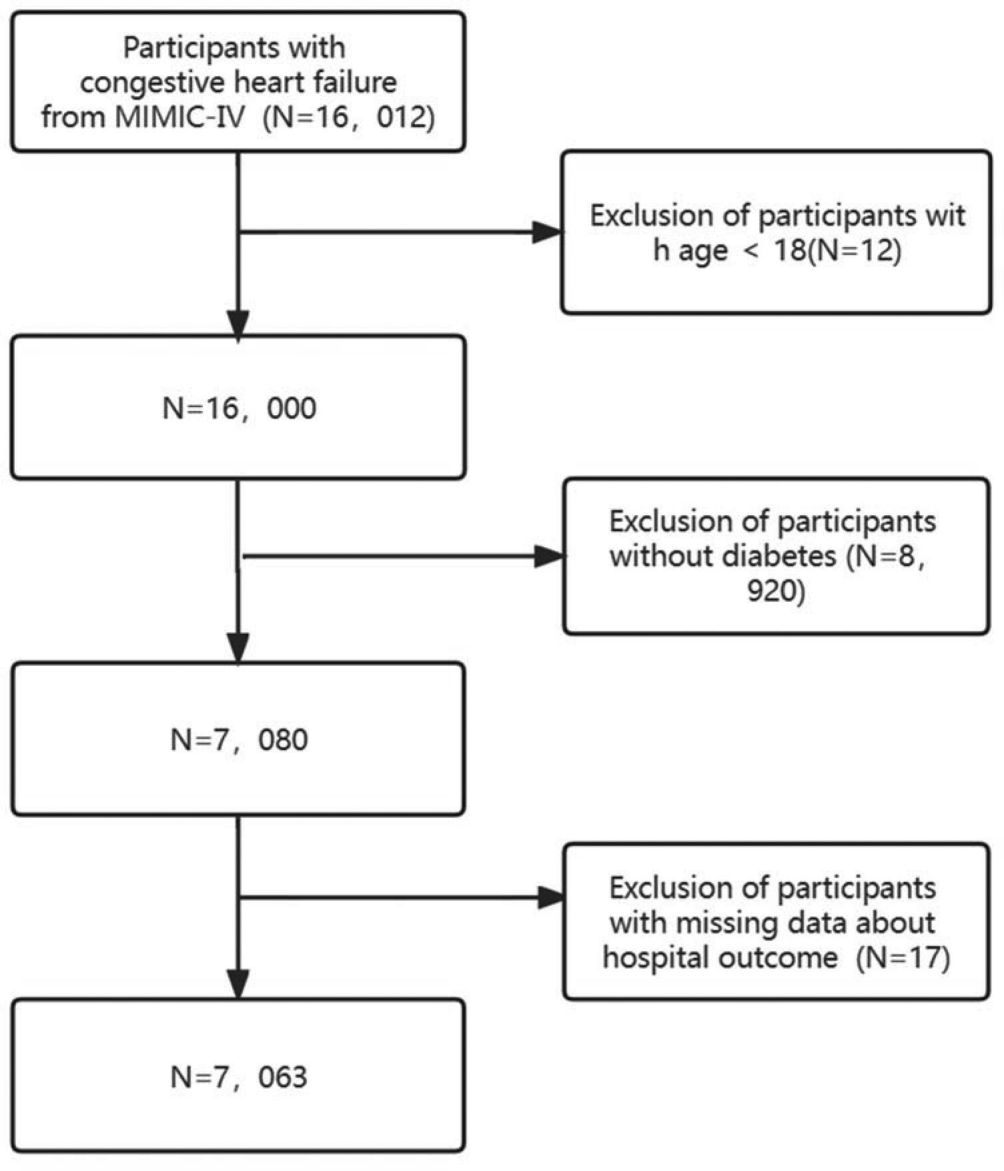
Flowchart of patient selection.

Identification of heart failure patients within the MIMIC-IV database employed ICD-9CM code 428, while diabetic patients were screened using ICD-9-CM code 250. Our analysis encompassed 16,012 retrospectively selected patients diagnosed with congestive heart failure (CHF), excluding those under 18 years of age (n = 12). Subsequent exclusions comprised patients with incomplete outcome data (n = 17) and non-diabetic patients (n = 8920). Ultimately, the final study cohort comprised 7063 patients after thorough screening, as depicted in Fig. 1.

### Data collection and primary outcome

The primary exposure of interest pertains to the serum calcium levels of patients. This serum calcium datum corresponds to the highest recorded value within the initial 24 h period subsequent to the patients’ admission to the intensive care unit (ICU), denominated in milligrams per deciliter (mg/dL). The principal outcome under investigation is the in-hospital mortality rate, denoting the proportion of patients who undergo fatal consequences due to a deterioration in their condition or other causes during a specific time frame while receiving treatment within hospitals.

We conducted a comprehensive data collection employing Structured Query Language (SQL), encompassing continuous variables such as age, anion gap (AG), blood urea nitrogen (BUN), chloride, creatinine, hemoglobin (Hb), mean corpuscular hemoglobin (MCH), mean corpuscular hemoglobin concentration (MCHC), mean corpuscular volume (MCV), platelet count, potassium, sodium, alongside severity scores represented by SOFA and APSIII. Additionally, we recorded other continuous variables pertaining to vital signs such as temperature and heart rate. A diverse array of categorical variables, including gender, race, complicating medical conditions, medication regimens, and medical procedures, were also collected. Subsequently, we selected the following four complicating medical conditions as covariates, drawing from analogous research: chronic obstructive pulmonary disease (COPD), acute myocardial infarction (AMI), melanosis coli (MC), and hepatic failure (HepF).

### Statistical analysis

Firstly, Table 1 presents baseline demographic and clinical data for the cohorts. Continuous variables with normal distributions are displayed as mean ± standard deviation (SD) and were compared using Student’s t-test or ANOVA. Skewed continuous variables are presented as medians ± interquartile ranges (IQR) and were analyzed with Mann–Whitney U or Kruskal–Wallis tests. Categorical variables were assessed for association using the chi-square test and reported as percentages. Differences among groups, stratified by serum calcium levels, were evaluated via one-way ANOVA or Kruskal–Wallis tests.

**Table 1:** Characteristics of the study population (N = 7063).

Variables	Total (n = 7063)	Q1 (n = 983)	Q2 (n = 827)	Q3 (n = 1118)	Q4 (n = 810)	Q5 (n = 1211)	Q6 (n = 963)	Q7 (n = 1151)	P value^a^
Age, mean ± SD	71.5 ± 12.2	71.5 ± 12.4	71.4 ± 12.1	72.0 ± 12.0	71.8 ± 12.1	71.7 ± 11.8	71.7 ± 12.0	70.7 ± 13.0	0.248
Gender, n (%)									< 0.001
Male	3928 (55.6)	587 (59.7)	484 (58.5)	661 (59.1)	439 (54.2)	650 (53.7)	537 (55.8)	570 (49.5)	
Female	3135 (44.4)	396 (40.3)	343 (41.5)	457 (40.9)	371 (45.8)	561 (46.3)	426 (44.2)	581 (50.5)	
Race, n (%)									< 0.001
White	4449 (63.0)	636 (64.7)	556 (67.2)	730 (65.3)	502 (62)	773 (63.8)	597 (62)	655 (56.9)	
Black	1275 (18.1)	139 (14.1)	115 (13.9)	149 (13.3)	126 (15.6)	240 (19.8)	208 (21.6)	298 (25.9)	
Other	1339 (19.0)	208 (21.2)	156 (18.9)	239 (21.4)	182 (22.5)	198 (16.4)	158 (16.4)	198 (17.2)	
Medication situation									
Norepinephrine, n (%)									< 0.001
No	5349 (75.7)	603 (61.3)	563 (68.1)	846 (75.7)	626 (77.3)	962 (79.4)	803 (83.4)	946 (82.2)	
Yes	1714 (24.3)	380 (38.7)	264 (31.9)	272 (24.3)	184 (22.7)	249 (20.6)	160 (16.6)	205 (17.8)	
Dopamine, n (%)									0.013
No	6831 (96.7)	934 (95)	794 (96)	1090 (97.5)	782 (96.5)	1178 (97.3)	940 (97.6)	1113 (96.7)	
Yes	232 ( 3.3)	49 (5)	33 (4)	28 (2.5)	28 (3.5)	33 (2.7)	23 (2.4)	38 (3.3)	
Epinephrine, n (%)									0.014
No	6728 (95.3)	925 (94.1)	778 (94.1)	1062 (95)	769 (94.9)	1150 (95)	934 (97)	1110 (96.4)	
Yes	335 (4.7)	58 (5.9)	49 (5.9)	56 (5)	41 (5.1)	61 (5)	29 (3)	41 (3.6)	
Phenylephrine, n (%)									< 0.001
No	6540 (92.6)	867 (88.2)	739 (89.4)	1031 (92.2)	759 (93.7)	1132 (93.5)	919 (95.4)	1093 (95)	
Yes	523 (7.4)	116 (11.8)	88 (10.6)	87 (7.8)	51 (6.3)	79 (6.5)	44 (4.6)	58 (5)	
Vasopressin, n (%)									< 0.001
No	6545 (92.7)	857 (87.2)	744 (90)	1039 (92.9)	760 (93.8)	1136 (93.8)	921 (95.6)	1088 (94.5)	
Yes	518 (7.3)	126 (12.8)	83 (10)	79 (7.1)	50 (6.2)	75 (6.2)	42 (4.4)	63 (5.5)	
Medical procedures									
Vent, n (%)									< 0.001
No	1044 (14.8)	135 (13.7)	103 (12.5)	136 (12.2)	108 (13.3)	203 (16.8)	145 (15.1)	214 (18.6)	
Yes	6019 (85.2)	848 (86.3)	724 (87.5)	982 (87.8)	702 (86.7)	1008 (83.2)	818 (84.9)	937 (81.4)	
Intubated, n (%)									< 0.001
No	4944 (70.0)	611 (62.2)	525 (63.5)	744 (66.5)	531 (65.6)	877 (72.4)	739 (76.7)	917 (79.7)	
Yes	2119 (30.0)	372 (37.8)	302 (36.5)	374 (33.5)	279 (34.4)	334 (27.6)	224 (23.3)	234 (20.3)	
Complicating disease									
COPD, n (%)									< 0.001
No	4321 (61.2)	655 (66.6)	509 (61.5)	716 (64)	490 (60.5)	740 (61.1)	542 (56.3)	669 (58.1)	
Yes	2742 (38.8)	328 (33.4)	318 (38.5)	402 (36)	320 (39.5)	471 (38.9)	421 (43.7)	482 (41.9)	
AMI, n (%)									0.106
No	4527 (64.1)	623 (63.4)	500 (60.5)	713 (63.8)	532 (65.7)	770 (63.6)	617 (64.1)	772 (67.1)	
Yes	2536 (35.9)	360 (36.6)	327 (39.5)	405 (36.2)	278 (34.3)	441 (36.4)	346 (35.9)	379 (32.9)	
MC, n (%)									0.004
No	6495 (92.0)	880 (89.5)	745 (90.1)	1036 (92.7)	744 (91.9)	1136 (93.8)	886 (92)	1068 (92.8)	
Yes	568 ( 8.0)	103 (10.5)	82 (9.9)	82 (7.3)	66 (8.1)	75 (6.2)	77 (8)	83 (7.2)	
HepF, n (%)									0.379
No	6878 (97.4)	950 (96.6)	803 (97.1)	1085 (97)	786 (97)	1184 (97.8)	942 (97.8)	1128 (98)	
Yes	185 ( 2.6)	33 (3.4)	24 (2.9)	33 (3)	24 (3)	27 (2.2)	21 (2.2)	23 (2)	
Vital signs									
Temperature, mean ± SD	36.8 ± 0.5	36.7 ± 0.7	36.8 ± 0.6	36.8 ± 0.5	36.8 ± 0.5	36.8 ± 0.5	36.8 ± 0.4	36.7 ± 0.5	< 0.001
Respiratory rate, mean ± SD	19.8 ± 3.7	19.7 ± 3.9	19.8 ± 3.6	19.6 ± 3.6	19.8 ± 3.7	19.8 ± 3.7	19.8 ± 3.6	19.9 ± 3.8	0.438
Heart rate, mean ± SD	83.3 ± 15.3	84.5 ± 16.3	84.8 ± 15.2	82.5 ± 14.6	83.1 ± 15.2	82.9 ± 15.7	82.9 ± 15.1	82.8 ± 15.2	0.003
SBP, mean ± SD	118.7 ± 17.7	114.3 ± 17.0	115.8 ± 16.7	118.4 ± 16.6	117.4 ± 16.5	119.8 ± 17.6	120.9 ± 17.3	122.5 ± 19.7	< 0.001
Blood biochemical indicators									
AG, mean ± SD	15.8 ± 4.6	15.6 ± 5.4	15.6 ± 4.7	15.2 ± 4.4	15.6 ± 4.3	15.6 ± 4.2	15.9 ± 4.1	17.1 ± 5.0	< 0.001
BUN, mean ± SD	41.3 ± 27.8	41.2 ± 28.5	40.5 ± 27.1	39.7 ± 28.2	39.6 ± 26.8	40.4 ± 26.5	41.9 ± 27.7	45.0 ± 29.1	< 0.001
Chloride, mean ± SD	101.6 ± 7.0	103.9 ± 7.7	102.8 ± 7.0	102.5 ± 6.8	101.4 ± 6.7	101.3 ± 6.5	100.4 ± 6.5	99.1 ± 6.7	< 0.001
Creatinine, mean ± SD	2.2 ± 1.9	2.4 ± 2.2	2.2 ± 1.9	2.1 ± 1.7	2.1 ± 1.8	2.1 ± 1.8	2.1 ± 1.7	2.6 ± 2.3	< 0.001
Hb, mean ± SD	10.0 ± 2.1	9.4 ± 1.9	9.6 ± 2.0	9.7 ± 2.0	10.0 ± 2.1	10.1 ± 2.0	10.4 ± 2.1	10.7 ± 2.2	< 0.001
MCH, mean ± SD	29.2 ± 2.8	29.4 ± 2.7	29.3 ± 2.7	29.3 ± 2.7	29.2 ± 2.6	29.1 ± 2.8	28.9 ± 2.9	29.1 ± 2.8	< 0.001
MCHC, mean ± SD	32.1 ± 1.8	32.2 ± 1.8	32.2 ± 1.7	32.2 ± 1.7	32.2 ± 1.7	32.1 ± 1.7	31.9 ± 1.8	32.0 ± 1.8	< 0.001
MCV, mean ± SD	90.9 ± 7.4	91.2 ± 7.4	91.1 ± 7.6	91.0 ± 7.4	90.5 ± 7.0	90.6 ± 7.3	90.5 ± 7.7	91.0 ± 7.6	0.183
Platelet, mean ± SD	211.8 ± 96.8	199.3 ± 99.6	206.3 ± 103.5	202.0 ± 90.6	208.3 ± 91.2	215.6 ± 92.6	222.4 ± 104.4	225.3 ± 93.9	< 0.001
Potassium, mean ± SD	4.4 ± 0.8	4.3 ± 0.9	4.4 ± 0.8	4.4 ± 0.8	4.4 ± 0.8	4.4 ± 0.8	4.5 ± 0.8	4.6 ± 0.9	< 0.001
Sodium, mean ± SD	137.9 ± 5.4	137.5 ± 6.0	137.4 ± 5.5	137.8 ± 5.4	137.8 ± 5.3	138.1 ± 5.0	138.3 ± 5.2	138.1 ± 5.3	< 0.001
RBC, mean ± SD	3.5 ± 0.8	3.2 ± 0.7	3.3 ± 0.7	3.3 ± 0.7	3.4 ± 0.7	3.5 ± 0.7	3.6 ± 0.8	3.7 ± 0.8	< 0.001
RDW, mean ± SD	16.0 ± 2.4	16.2 ± 2.3	16.0 ± 2.3	15.9 ± 2.4	15.9 ± 2.4	15.9 ± 2.2	16.0 ± 2.4	16.0 ± 2.4	0.075
WBC, mean ± SD	11.7 ± 8.1	12.6 ± 8.2	13.4 ± 11.7	11.7 ± 6.7	11.5 ± 7.4	11.1 ± 6.2	11.4 ± 9.8	10.9 ± 6.3	< 0.001
Hstatus, n (%)									< 0.001
0	6203 (87.8)	803 (81.7)	704 (85.1)	1003 (89.7)	730 (90.1)	1067 (88.1)	889 (92.3)	1007 (87.5)	
1	860 (12.2)	180 (18.3)	123 (14.9)	115 (10.3)	80 (9.9)	144 (11.9)	74 (7.7)	144 (12.5)	
SOFA, mean ± SD	3.6 ± 2.9	4.4 ± 3.2	4.1 ± 3.1	3.7 ± 3.0	3.4 ± 2.9	3.3 ± 2.9	3.1 ± 2.6	3.2 ± 2.9	< 0.001
APSIII, mean ± SD	54.5 ± 21.7	61.6 ± 24.3	57.0 ± 22.0	53.9 ± 21.4	53.4 ± 21.2	52.3 ± 21.1	50.6 ± 18.6	53.6 ± 21.2	< 0.001

Q1(≤ 7.7 mg/dL), Q2(7.7–8 mg/dL) Q3(8–8.3 mg/dL) Q4(8.3–8.5 mg/dL) Q5(8.5–8.8 mg/dL) Q6(8.8–9.1 mg/dL) Q7(≥ 9.1 mg/dL).

*%* weighted proportion, *Hstatus* hospital status, *CHF* congestive heart failure, *COPD* chronic obstructive pulmonary disease, *HepF* hepatic failure, *AMI* acute myocardial infarction, *APSIII* Acute Physiology III, *SOFA* Sequential Organ Failure Assessment, *SBP* systolic blood pressure, *AG* anion gap, *BUN* blood urea nitrogen, *MCH* mean corpuscular hemoglobin, *MCHC* mean corpuscular hemoglobin concentration, *MCV* mean corpuscular volume, *RBC* red blood cell, *RDW* red blood cell distribution width, *WBC* white blood cell count.

^a^P values of multiple comparisons were corrected by the False Discovery Rate method.

^b^Q1-Q7: according to serum calcium.

Secondly, to mitigate potential confounding, multivariate logistic regression examined the link between blood calcium levels and in-hospital mortality across seven groups. Odds ratios (ORs) and 95% confidence intervals (CIs) were calculated. Stepwise regression models were utilized, progressively adjusting for covariates. Model 1 was unadjusted, while Model 2 incorporated demographic variables (gender, age, race). Model 3 added complicating diseases (COPD, AMI, MC, HepF), and Model 4 further adjusted for medical procedures, medication usage, vital signs, and blood biochemical indicators. Model 5 included APSIII and SOFA scores. Linear trend assessments were conducted using categorical variables as continuous in regression models.

Furthermore, to examine the dose–response relationship between serum calcium and in-hospital mortality, cubic spline and penalized spline models were fitted. Threshold effects of serum calcium on mortality were analyzed using piecewise regression models.

Lastly, subgroup analyses depicted prognostic associations between serum calcium and patient characteristics, presented through forest plots. Forest plots described subgroup analyses including age, gender, race, and presence of COPD, AMI, and MC.

All analyses utilized R version 4.1.1 (R Foundation for Statistical Computing, Vienna, Austria) and Free Statistics software version 1.7. Statistical significance was set at P < 0.05 (two-sided). Reporting adhered to the Strengthening the Reporting of Observational Studies in Epidemiology (STROBE) statement for studies.

### Ethics approval and consent to participate

The establishment of this database was approved by the Massachusetts Institute of Technology (Cambridge, MA, USA) and Beth Israel Deaconess Medical Center (Boston, MA, USA), and informed consents were exempted due to all patients’ data were anonymized before the data were obtained. We also complied with all relevant ethical regulations regarding the use of the data in our study. All reports adhered to the guidelines for Strengthening the Reporting of Observational Studies in Epidemiology and the Declaration of Helsinki.

## Results

### Baseline characteristics of selected participants

The study enrolled a total of 7063 patients after a rigorous screening process (Fig. 1). Table 1 presents an overview of the baseline characteristics of the study population based on seven distinct subgroups categorized by serum calcium concentrations. Q1(≤ 7.7 mg/dL), Q2(7.7–8 mg/dL), Q3(8–8.3 mg/dL), Q4(8.3–8.5 mg/dL), Q5(8.5–8.8 mg/dL), Q6(8.8–9.1 mg/dL), Q7(≥ 9.1 mg/dL). The median age of the patients included in the study was 71.5 ± 12.2 years, with 55.6% male. Most participants were of white ethnicity, constituting 63% of the sample. Black participants constituted approximately 18.1%, while individuals from other racial backgrounds, including Asians, comprised 19% of the cohort. The overall in-hospital mortality rate stood at 12.2%.

Patients with elevated serum calcium levels displayed a higher proportion of female and black ethnicity, a greater incidence of COPD complications, elevated SBP, anion AG, Hb, platelet count, potassium, sodium, and red blood cell counts compared to patients with lower serum calcium levels. Conversely, the subgroup with decreased serum calcium levels exhibited an increased likelihood of requiring phenylephrine and vasopressin interventions, intubation, and ventilation. Additionally, this subgroup presented higher levels of chloride, MCH, WBC, SOFA scores, and APSIII scores. Blood biochemistry analyses indicated more pronounced BUN and Creatinine concentrations in both high and low calcium groups, with the most significant values observed in the highest calcium group, Q7. Similarly, deviations in serum calcium levels, whether elevated or diminished, correlated with heightened in-hospital mortality rates. Remarkably, the subgroup with the lowest serum calcium value displayed the highest in-hospital mortality rate of 18.3%. Detailed baseline characteristics are provided in Table 1.

### Relationship between serum calcium and in-hospital mortality

Table 2 depicts the correlation between serum calcium levels and in-hospital mortality among diabetes patients afflicted with congestive heart failure. Employing a multivariate logistic regression model, we computed the odds ratios (OR) accompanied by their corresponding 95% confidence intervals (CI). The adjustment for this study encompassed demographic characteristics, comorbidities, medical interventions, medication profiles, essential vital signs, blood biochemical markers, APSIII score, and SOFA score.

**Table 2:** Multivariable logistic regression to assess the association of serum calcium with In-hospital mortality rate.

Serum calcium	Model 1		Model 2		Model 3		Model 4		Model 5	
	OR_95 CI	P value	OR_95 CI	P value	OR_95CI	P value	OR_95CI	P value	OR_95CI	P value
Categorical variable										
Q1(≤ 7.7)	2.69 (2.02 ~ 3.59)	< 0.001	2.69 (2.01 ~ 3.6)	< 0.001	2.65 (1.98 ~ 3.55)	< 0.001	2.14 (1.5 ~ 3.05)	< 0.001	1.69 (1.17 ~ 2.44)	0.005
Q2(7.7–8)	2.1 (1.55 ~ 2.85)	< 0.001	2.12 (1.56 ~ 2.88)	< 0.001	2.07 (1.52 ~ 2.82)	< 0.001	1.79 (1.24 ~ 2.58)	0.002	1.62 (1.11 ~ 2.36)	0.013
Q3(8–8.3)	1.38 (1.01 ~ 1.87)	0.04	1.34 (0.98 ~ 1.82)	0.064	1.34 (0.98 ~ 1.82)	0.064	1.42 (0.99 ~ 2.02)	0.054	1.26 (0.87 ~ 1.83)	0.216
Q4(8.3–8.5)	1.32 (0.95 ~ 1.83)	0.103	1.29 (0.92 ~ 1.8)	0.139	1.28 (0.91 ~ 1.78)	0.154	1.26 (0.86 ~ 1.85)	0.232	1.15 (0.77 ~ 1.72)	0.486
Q5(8.5–8.8)	1.62 (1.21 ~ 2.18)	0.001	1.64 (1.22 ~ 2.2)	0.001	1.65 (1.23 ~ 2.22)	0.001	1.83 (1.31 ~ 2.57)	< 0.001	1.74 (1.22 ~ 2.48)	0.002
Q6(8.8–9.1)	1(Ref)		1(Ref)		1(Ref)		1(Ref)		1(Ref)	
Q7(≥ 9.1)	1.72 (1.28 ~ 2.31)	< 0.001	1.79 (1.33 ~ 2.41)	< 0.001	1.82 (1.35 ~ 2.45)	< 0.001	1.72 (1.22 ~ 2.42)	0.002	1.57 (1.1 ~ 2.24)	0.012
P for tread		< 0.001		< 0.001		< 0.001		0.043		0.373

Model 1: No adjustment.

Model 2: Adjusted for demographic variables (sex, age, race).

Model 3: Adjusted for demographic variables, Concomitant disease (COPD, AMI, MC, HepF).

Model 4: Adjusted for demographic variables, complicating disease, Medical Procedures (Vent, Intubated), Medication situation (Norepinephrine Dopamine Epinephrine Phenylephrine Vasopressin), Basic vital signs (Temperature Respiratory Rate Heart Rate SBP), Blood biochemical indicators (AG BUN Chloride Creatinine, Hb MCH MCHC MCV Platelet Potassium Sodium RBC RDW WBC).

Model 5: Adjusted for demographic variables, complicating disease, Medical Procedures, Medication situation, Basic vital signs, Blood biochemical indicators, APSIII, SOFA.

The unit of calcium is mg/dl.

*%* weighted proportion, *CHF* congestive heart failure, *COPD* chronic obstructive pulmonary disease, *HepF* hepatic failure, *AMI* acute myocardial infarction, *APSIII* Acute Physiology III, *SOFA* Sequential Organ Failure Assessment, *SBP* systolic blood pressure, AG anion gap, *BUN* blood urea nitrogen, *MCH* mean corpuscular hemoglobin, *MCHC* mean corpuscular hemoglobin concentration, *MCV* mean corpuscular volume, *RBC* red blood cell, *RDW* red blood cell distribution width, *WBC* white blood cell count, *CI* confidence interval, *OR* odds ratios, *Ref* reference.

In the unadjusted analysis, the cohorts displaying the lowest serum calcium levels (Q1 and Q2) exhibited a notably heightened susceptibility to in-hospital mortality, reflecting a 169% increase (OR = 2.69, 95% CI 2.02–3.59, p < 0.001) and a 110% increase (OR = 2.1, 95% CI 1.55–2.85, p < 0.001), respectively, in contrast to the reference group. Similarly, the high serum calcium group demonstrated a comparable trend, indicating a 72% elevated risk of in-hospital mortality within Q7 (OR = 1.72, 95% CI 1.28–2.31, p < 0.001).

It is noteworthy that this observed association retained its statistical significance even subsequent to comprehensive covariate adjustment. Specifically, following full adjustment, Q1 exhibited a 69% elevation in in-hospital mortality (OR = 1.69, 95% CI 1.17–2.44, p = 0.005), while Q2 demonstrated a 62% increase in in-hospital mortality (OR = 1.62, 95% CI 1.11–2.36, p = 0.013). Furthermore, the adjusted OR for Q7 stood at 1.57 (95% CI 1.1–2.24, p = 0.012).

### Dose–response relationships

**Figure 2:**
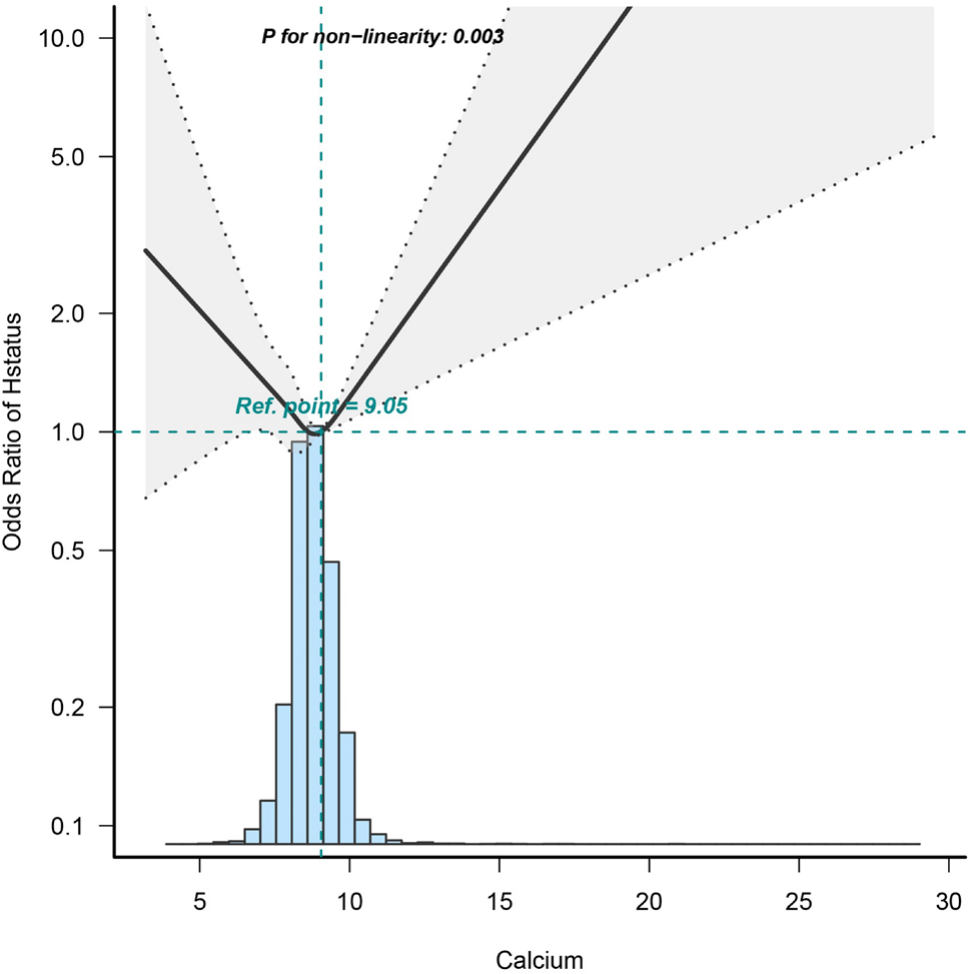
Dose–Response Relationships between Blood calcium with In-hospital mortality odds ratio. Solid and dashed lines represent the predicted value and 95% confidence intervals. Adjusted for demographic variables (sex, age, race), Concomitant disease(COPD,AMI,MC, HepF), Medical Procedures(Vent, Intubated), Medication situation(Norepinephrine Dopamine Epinephrine Phenylephrine Vasopressin),Basic vital signs(Temperature Respiratory Rate Heart Rate SBP),Blood biochemical indicators(AG BUN Chloride Creatinine Hb MCH MCHC MCV Platelet Potassium Sodium RBC RDW WBC), APSIII,SOFA. Only 99% of the data is shown. *%* weighted proportion, *CHF* congestive heart failure, *COPD* chronic obstructive pulmonary disease, *HepF* hepatic failure, *AMI* acute myocardial infarction, *APSIII* Acute Physiology III, *SOFA* Sequential Organ Failure Assessment, *SBP* systolic blood pressure, AG anion gap, *BUN* blood urea nitrogen, *MCH* mean corpuscular hemoglobin, *MCHC* mean corpuscular hemoglobin concentration, *MCV* mean corpuscular volume, *RBC* red blood cell, *RDW* red blood cell distribution width, *WBC* white blood cell count, *CI* confidence interval, *OR* odds ratios, *Ref* reference.

The dose–response analysis, as shown in Fig. 2 (P for nonlinearity = 0.03), unveiled a non-linear link between serum calcium levels and in-hospital mortality, following adjustments for potential covariates. The spline curves vividly portray the U-shaped trend characterizing this relationship. Moreover, the study identified a threshold effect in the nexus between serum calcium levels and in-hospital mortality, as highlighted in Table 3. Among patients exhibiting serum calcium levels ≤ 9.05 mg/dL, the odds ratio (OR) for in-hospital mortality stood at 0.814 (95% CI 0.678–0.976; p = 0.0262), denoting an 18.6% reduction in the relative risk of in-hospital mortality with each unit increase in serum calcium levels. Conversely, beyond the inflection point of 9.05 mg/dL, a subtle positive correlation emerged linking serum calcium levels to in-hospital mortality (OR = 1.153, 95% CI 0.818–1.626, p = 0.4158).

**Table 3:** Threshold effect analysis of relationship of serum calcium with In-hospital mortality rate.

	Adjusted OR_95CI	P value
Two model		
Serum calcium ≤ 9.05 mg/dL	0.814 (0.678 ~ 0.976)	0.0262
Serum calcium ≥ 9.05 mg/dL	1.153 (0.818 ~ 1.626)	0.4158
Likelihood ratio test	–	0.044

Adjusted for demographic variables (sex, age, race), Concomitant disease (COPD,AMI,MC, HepF), Medical Procedures (Vent, Intubated), Medication situation (Norepinephrine Dopamine Epinephrine Phenylephrine Vasopressin), Basic vital signs (Temperature Respiratory Rate Heart Rate SBP), Blood biochemical indicators (AG BUN Chloride Creatinine Hb MCH MCHC MCV Platelet Potassium Sodium RBC RDW WBC), APSIII,SOFA.

*%* weighted proportion, *CHF* congestive heart failure, *COPD* chronic obstructive pulmonary disease, *HepF* hepatic failure, *AMI* acute myocardial infarction, *APSIII* Acute Physiology III, *SOFA* Sequential Organ Failure Assessment, *SBP* systolic blood pressure, AG anion gap, *BUN* blood urea nitrogen, *MCH* mean corpuscular hemoglobin, *MCHC* mean corpuscular hemoglobin concentration, *MCV* mean corpuscular volume, *RBC* red blood cell, *RDW* red blood cell distribution width, *WBC* white blood cell count, *CI* confidence interval, *OR* odds ratios, *Ref* reference.

### Subgroup analysis

We performed subgroup analyses to investigate potential modifications of this association by other confounding variables (Supplement Fig. 1). The subgroup analyses incorporated stratification variables such as age, gender, COPD, AMI, and MC. No significant interaction was observed between in-hospital mortality and all of the stratification above variables (p > 0.05), further confirming the reliability of our conclusions.

## Discussion

Numerous studies have identified prevalent disturbances in serum calcium homeostasis among critically ill patients, wherein both elevated and diminished levels correlate with heightened susceptibility to adverse clinical outcomes^24,25^. This phenomenon has been independently corroborated in cohorts of patients with diabetes and heart failure^16,17,19^. To the best of our knowledge, we are the first to demonstrate such an association between blood calcium concentrations and in-hospital mortality in the context of concomitant diabetes mellitus and heart failure. Our findings distinctly unveil a substantial nexus between in-hospital mortality and aberrant serum calcium levels, encompassing both hypercalcemia and hypocalcemia, within individuals afflicted with diabetes mellitus and heart failure. Importantly, this correlation perseveres even subsequent to meticulous adjustment for plausible confounding variables.

In alignment with our investigation, prior research has consistently indicated a robust correlation between serum calcium levels and the onset, prognosis, and outcomes of individuals afflicted with diabetes or heart failure. A recent cross-sectional study, employing a constrained cubic spline curve, unveiled a J-shaped association between serum calcium levels and type 2 diabetes (T2D). Logistic regression analysis further disclosed a statistically significant elevation in the risk of T2D among individuals in the high serum calcium group in comparison to the moderate group^26^. Concurrently, three distinct cohort analyses conducted in China (n = 6096)^27^, the United States (n = 12,800)^28^, and Norway (n = 27,158)^29^, consistently affirmed a positive correlation between serum calcium levels and diabetes risk. Moreover, serum calcium exhibits an indirect influence on in-hospital mortality among diabetic patients. Kwak et al., by introducing serum calcium as a continuous variable in a multivariate model, demonstrated that each 1 SD mg increase in serum calcium resulted in a 30% rise in coronary artery calcification scores^16^. A significant interaction between the average coronary calcium score (CCS) and diabetes mellitus (DM) also emerged (p < 0.00001). Notably, mortality rates exhibited a substantial elevation in diabetic subjects relative to non-diabetic subjects as CCS levels escalated^17^. These findings collectively suggest a connection between hypercalcemia and unfavorable outcomes among diabetic individuals, partially aligning with our own observations. However, existing conclusions primarily emphasize high serum calcium as a diabetes risk factor, with insufficient consideration for the impact of low serum calcium on mortality. This discordance underscores the intricate nature of the relationship between calcium levels and diabetes, likely influenced by factors such as sample size, ethnicity, confounding adjustments, and study design. While studies pertaining to heart failure patients are comparatively smaller than those concentrating on diabetes, a cross-sectional echocardiographic investigation identified an independent association between elevated albumin-adjusted serum calcium levels and heart failure with preserved ejection fraction in individuals with type 2 diabetes mellitus^30^. Notably congruent with our findings, a Danish cohort study involving chronic heart failure patients revealed a parallel observation. Both hypocalcemia and hypercalcemia conferred increased mortality risks in the short term (within 30 days) and the long term (beyond 30 days)^19^.

In our study, we observed a U-shaped dose–response relationship between serum calcium levels and the odds ratio for intrahospital mortality. This curve exhibited an initial decrease followed by an increase. The inflection point of the curve, corresponding to the nadir, occurred around 9.05 mg/dL. At this point, the odds ratio for in-hospital mortality reached 1. Deviations from the 9.05 mg/dL threshold, whether higher or lower, were associated with an escalating risk of in-hospital mortality. Previous research has predominantly focused on heart failure or diabetes as individual ailments. However, given the frequent coexistence of these conditions, our study design better reflects real-world clinical scenarios. Moreover, our investigation underscores the importance of hypocalcemia as a critical indicator warranting meticulous attention. Within a clinical context, low serum calcium levels hold as much significance as elevated levels. The findings of this study hold the potential to facilitate earlier and more efficacious medical interventions and strategies for individuals dealing with both diabetes mellitus and heart failure, ultimately culminating in a reduction of their mortality rate.

The precise mechanism underlying the association between serum calcium and the concurrent diagnosis of diabetes mellitus (DM) and congestive heart failure (CHF) remains unclear, though several potential hypotheses have been proposed. Firstly, calcium influx is implicated in the stimulation of insulin secretion, and its aberration is linked to β-cell dysfunction and the onset of type 2 diabetes^31^. Additionally, calcium ions (Ca2 +) modulate insulin signaling pathways, thereby influencing cellular insulin sensitivity^32^. Research suggests that heightened serum calcium levels inhibit the production of parathyroid hormone (PTH), leading to a reduction in the biologically active form of vitamin D^33^. Deficiency in vitamin D augments systemic inflammatory mediators, exacerbates cellular oxidative stress, suppresses the immune system’s release of anti-inflammatory factors, and heightens susceptibility to diabetes and cardiovascular disease^34–36^. Furthermore, a complex interplay exists between cardiovascular well-being and mineral homeostasis. Calcium functions as a pivotal secondary messenger, exerting a critical role in governing mitochondrial activity and upholding excitation–contraction coupling mechanisms^37^. Perturbation of calcium homeostasis detrimentally impacts the contractile proficiency of the heart, culminating in compromised cardiac performance^38^. Anomalies in calcium signaling may lead to cardiac arrhythmias, thereby exacerbating heart failure conditions^39^. The deleterious effects of calcium imbalance extend to myocardial and vascular structures. For instance, the deposition of calcium along the coronary artery walls gives rise to coronary calcification, a well-recognized risk factor contributing to the progression of heart failure^40,41^.

Our research possesses notable design and statistical advantages. Initially, we focused on assessing serum calcium levels, a clinically feasible metric. Calcium’s role as a straightforward and dependable clinical marker enhances the translational prospects of our theoretical discoveries in the realm of medical application. Secondly, our investigation represents a pioneering effort in examining a sizable cohort of patients with diabetes and heart failure, distinguished by a substantial sample size and data quality. Furthermore, our study substantiates a significant non-linear correlation between serum calcium levels and in-hospital mortality, demonstrated via refined curve fitting techniques. Additionally, we mitigated bias arising from confounding variables by employing subgroup analyses and multivariate logistic regression.

However, there exist several limitations within this study. First, it is a retrospective cohort study, which by design cannot establish causal relationships, but is intended to assess associations. Secondly, our methodology used ICD9 codes to identify patients. But the method relies on the accuracy of ICD9 coding. Coding inconsistencies vary significantly by diagnosis, and rates of coding issues have been reported as high as 80%^42^. Thirdly, different types of heart failure, such as Heart failure with preserved ejection fraction (HFpEF), Heart failure with reduced ejection fraction (HFrEF), and Heart failure with mildly reduced ejection fraction (HFmrEF), may have different impacts on the results. However, due to the lack of relevant subtype heart failure data, we will verify it in our own database in the future. In addition, our study population primarily consisted of participants in the United States, which may limit the generalization of the findings to other populations. Finally, due to the limitations of the institutional database, we were unable to collect detailed information on different drugs in patients, which plays an important role in heart failure (HF). For example, different types and doses of diuretics, patient enuresis status, and patient resistance to diuretics have a certain impact on the treatment and management of heart failure in patients. And the role of parathyroid hormone is related to calcium serum levels and prognosis of patients with HF^43^. Similarly, we used total serum calcium level rather than corrected values of calcium because it is readily available. In the future, based on the present research, we will further supplement the data and conduct detailed and conduct prospective controlled trials with large sample sizes to provide more powerful evidence to support our findings.

In summary, our study expands upon prior research concerning the links between serum calcium and diabetes as well as heart failure. Deviations in serum calcium levels, whether elevated or diminished, significantly impact the likelihood of in-hospital mortality among diabetes patients with congestive heart failure. Optimal in-hospital survival rates were observed when serum calcium levels reached 9.05 mg/dL. The serum calcium level holds promise as a straightforward and dependable prognostic marker.

### Supplementary Information

Supplementary Figure 1.

## Data Availability

The datasets used and analyzed during the current study are available from the corresponding author upon reasonable request. To obtain the application executable files, please contact the author Kai Zhang by email zhangkai7018@jlu.edu.cn.

## References

[1] Zimmet P (2016). Diabetes mellitus statistics on prevalence and mortality: Facts and fallacies. Nat. Rev. Endocrinol..

[2] Federation, I.D. *IDF Diabetes Atlas 10th edn*. http://www.diabetesatlas.org. (2021).

[3] McMurray JJ (2014). Heart failure: A cardiovascular outcome in diabetes that can no longer be ignored. Lancet Diabetes Endocrinol..

[4] Gilbert RE, Krum H (2015). Heart failure in diabetes: Effects of anti-hyperglycaemic drug therapy. Lancet.

[5] Sarma S (2013). Association between diabetes mellitus and post-discharge outcomes in patients hospitalized with heart failure: Findings from the EVEREST trial. Eur. J. Heart Fail..

[6] Zhang F (2021). Resveratrol pretreatment improved heart recovery ability of hyperglycemic bone marrow stem cells transplantation in diabetic myocardial infarction by down-regulating MicroRNA-34a. Front. Pharmacol..

[7] MacDonald MR (2008). Impact of diabetes on outcomes in patients with low and preserved ejection fraction heart failure: An analysis of the Candesartan in heart failure: Assessment of reduction in mortality and morbidity (CHARM) programme. Eur. Heart J..

[8] Kristensen SL (2016). Risk related to pre-diabetes mellitus and diabetes mellitus in heart failure with reduced ejection fraction: Insights from prospective comparison of ARNI with ACEI to determine impact on global mortality and morbidity in heart failure trial. Circ. Heart Fail..

[9] Hippisley-Cox J, Coupland C (2016). Diabetes treatments and risk of heart failure, cardiovascular disease, and all cause mortality: Cohort Study in primary care. Bmj.

[10] Hou Y (2022). New clusters of serum electrolytes aid in stratification of diabetes and metabolic risk. J. Diabetes.

[11] Milionis HJ (2002). Hypomagnesemia and concurrent acid-base and electrolyte abnormalities in patients with congestive heart failure. Eur. J. Heart Fail..

[12] Guan B (2016). GCM2-Activating mutations in familial isolated hyperparathyroidism. Am. J. Hum. Genet..

[13] Moe SM (2016). Calcium homeostasis in health and in kidney disease. Compr. Physiol..

[14] Baird GS (2011). Ionized calcium. Clin. Chim. Acta.

[15] Lorenzo C (2014). Calcium and phosphate concentrations and future development of type 2 diabetes: The Insulin Resistance Atherosclerosis Study. Diabetologia.

[16] Kwak SM (2014). Dietary intake of calcium and phosphorus and serum concentration in relation to the risk of coronary artery calcification in asymptomatic adults. Arterioscler. Thromb. Vasc. Biol..

[17] Raggi P (2004). Prognostic value of coronary artery calcium screening in subjects with and without diabetes. J. Am. Coll. Cardiol..

[18] Li J (2016). Association of serum calcium and heart failure with preserved ejection fraction in patients with type 2 diabetes. Cardiovasc. Diabetol..

[19] Jensen AC (2019). The association between serum calcium levels and short-term mortality in patients with chronic heart failure. Am. J. Med..

[20] Johnson AEW (2023). MIMIC-IV, a freely accessible electronic health record dataset. Sci. Data.

[21] Alistair Johnson, L.B., Tom Pollard, Steven Horng, Leo Anthony Celi, Roger Mark. *MIMIC-IV (version 1.0)*. PhysioNet 2021. 10.13026/s6n6-xd98.

[22] Tang Y (2021). Association of systemic immune-inflammation index with short-term mortality of congestive heart failure: A retrospective cohort study. Front. Cardiovasc. Med..

[23] Yang R (2022). The use of antibiotics for ventilator-associated pneumonia in the MIMIC-IV database. Front. Pharmacol..

[24] Akirov A (2017). Calcium levels on admission and before discharge are associated with mortality risk in hospitalized patients. Endocrine.

[25] Thongprayoon C (2020). Impact of changes in serum calcium levels on in-hospital mortality. Medicina (Kaunas).

[26] Zhai Z (2023). Association between serum calcium level and type 2 diabetes: An NHANES analysis and Mendelian Randomization Study. Diabet. Med..

[27] Sing CW (2016). Serum calcium and incident diabetes: An Observational Study and meta-analysis. Osteoporos. Int..

[28] Rooney MR (2016). Serum calcium and incident type 2 diabetes: The Atherosclerosis Risk in Communities (ARIC) study. Am. J. Clin. Nutr..

[29] Jorde R (2013). Serum calcium and the calcium-sensing receptor polymorphism rs17251221 in relation to coronary heart disease, type 2 diabetes, cancer and mortality: The Tromsø study. Eur. J. Epidemiol..

[30] Helte E, Åkesson A, Larsson SC (2019). Assessing causality in associations of serum calcium and magnesium levels with heart failure: A two-sample Mendelian randomization study. Front. Genet..

[31] Idevall-Hagren O, Tengholm A (2020). Metabolic regulation of calcium signaling in beta cells. Semin. Cell Dev. Biol..

[32] Zemel MB (1998). Nutritional and endocrine modulation of intracellular calcium: Implications in obesity, insulin resistance and hypertension. Mol. Cell Biochem..

[33] Gil Á, Plaza-Diaz J, Mesa MD (2018). Vitamin D: Classic and novel actions. Ann. Nutr. Metab..

[34] Renke G (2023). Effects of vitamin D on cardiovascular risk and oxidative stress. Nutrients.

[35] Pittas AG (2007). The role of vitamin D and calcium in type 2 diabetes. A systematic review and meta-analysis. J. Clin. Endocrinol. Metab..

[36] Holick MF (2005). Vitamin D: Important for prevention of osteoporosis, cardiovascular heart disease, type 1 diabetes, autoimmune diseases, and some cancers. South Med. J..

[37] Chaanine AH (2021). Metabolic remodeling and implicated calcium and signal transduction pathways in the pathogenesis of heart failure. Int. J. Mol. Sci..

[38] Deus AF (2019). Myocardial dysfunction after severe food restriction is linked to changes in the calcium-handling properties in rats. Nutrients.

[39] Yang X (2015). Genetic deletion of Rnd3/RhoE results in mouse heart calcium leakage through upregulation of protein kinase a signaling. Circ. Res..

[40] Pagliaro BR (2020). Myocardial ischemia and coronary disease in heart failure. Heart Fail. Rev..

[41] Mori H (2018). Coronary artery calcification and its progression: What does it really mean?. JACC Cardiovasc. Imaging.

[42] Muntner P (2019). Measurement of blood pressure in humans: A scientific statement from the American heart association. Hypertension.

[43] Scicchitano P (2022). Plasma levels of intact parathyroid hormone and congestion burden in heart failure: Clinical correlations and prognostic role. J. Cardiovasc. Dev. Dis..

