# Supplementary material for: U-shaped association between serum calcium and in-hospital mortality in diabetes patients with congestive heart failure: a cohort study
Source: Sci Rep. 2024 Jun 11;14:13412. doi: 10.1038/s41598-024-63603-w (PMC11167038; doi:10.1038/s41598-024-63603-w)
Supplement: Supplementary file 1 — Supplementary Figure 1. [file 41598_2024_63603_MOESM1_ESM.docx]

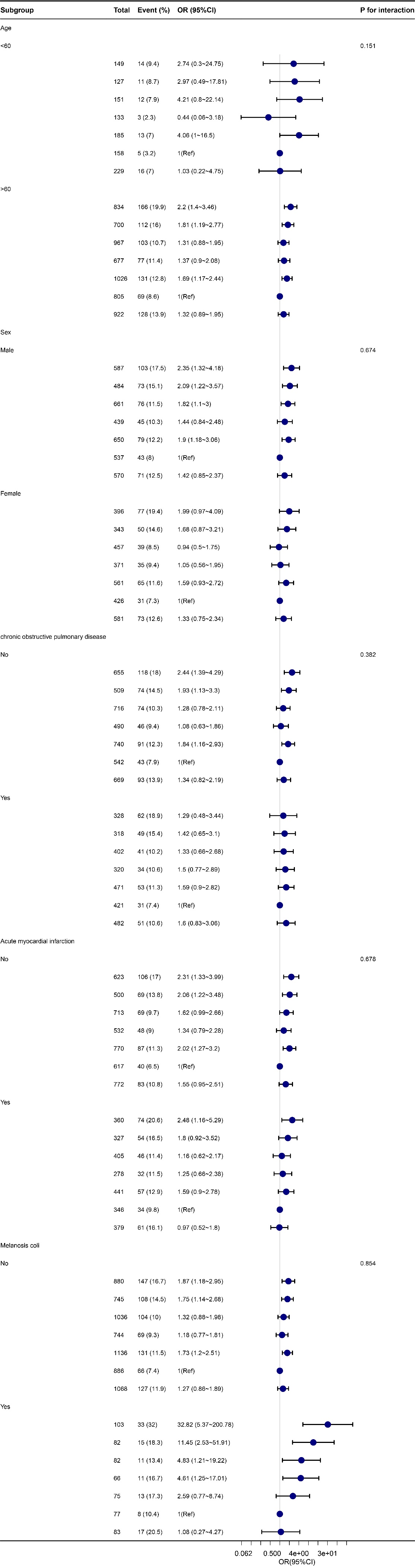


**Supplement Figure.** Stratified analyses of the association between Blood calcium with In-hospital mortality rate.

Note: The p value for interaction represents the likelihood of interaction between the Blood calcium with Intrahospital mortality rate.

Abbreviations: OR, odd ratio; CI, confidence interval.
